# Isolation and characterization of intestinal bacteria associated with cellulose degradation in grasshoppers (Orthoptera)

**DOI:** 10.1093/jisesa/iead101

**Published:** 2023-11-24

**Authors:** Wen-Jing Li, Fei-Fei Li, Jing Bai, Ke Liang, Kai Li, Guo-Qing Qin, Yu-Long Zhang, Xin-Jiang Li

**Affiliations:** The Key Laboratory of Zoological Systematics and Application, School of Life Sciences, Institute of Life Sciences and Green Development, Hebei University, Baoding, China; The Key Laboratory of Zoological Systematics and Application, School of Life Sciences, Institute of Life Sciences and Green Development, Hebei University, Baoding, China; The Key Laboratory of Zoological Systematics and Application, School of Life Sciences, Institute of Life Sciences and Green Development, Hebei University, Baoding, China; The Key Laboratory of Zoological Systematics and Application, School of Life Sciences, Institute of Life Sciences and Green Development, Hebei University, Baoding, China; The Key Laboratory of Zoological Systematics and Application, School of Life Sciences, Institute of Life Sciences and Green Development, Hebei University, Baoding, China; The Key Laboratory of Zoological Systematics and Application, School of Life Sciences, Institute of Life Sciences and Green Development, Hebei University, Baoding, China; The Key Laboratory of Zoological Systematics and Application, School of Life Sciences, Institute of Life Sciences and Green Development, Hebei University, Baoding, China; The Key Laboratory of Zoological Systematics and Application, School of Life Sciences, Institute of Life Sciences and Green Development, Hebei University, Baoding, China

**Keywords:** grasshopper, cellulose degradation rate, *Klebsiella*, *Aeromonas*, *Bacillus*

## Abstract

Insect gut bacteria play an essential role in the nutritional metabolism, growth, and development of insects. Grasshoppers (Orthoptera) are cellulose-rich plant-feeding pests. Although the biological potential of grasshopper gut microorganisms to assist cellulose decomposition is well established, microbial resources for efficient degradation of cellulose biomass are still scarce and need to be developed. In this study, we used selective media to isolate cellulose-degrading bacteria from the intestines of *Atractomorpha sinensis, Trilophidia annulata, Sphingonotus mongolicus*, and *Calliptamus abbreviatus.* Phylogenetic analysis based on the maximum likelihood method using 16S rDNA sequencing sequences to identify bacteria revealed the isolation of 11 strains belonging to 3 genera, including *Klebsiella*, *Aeromonas*, and *Bacillus*. The degradability of the isolates to cellulose was then determined by the DNS colorimetric method, and the results showed that *Bacillus* had the highest degradation rate. The elucidation of microbial cellulose degradation capacity in grasshoppers not only contributes to the understanding of multiple plant–insect–microbe interactions, but also provides a valuable microbial resource for solving the biomass conversion of cellulose species problem.

## Introduction

Cellulose is the polysaccharide substance most widely distributed and abundant in nature and is a renewable natural macromolecular compound, with glucose as the final product of digestion and decomposition (Huang et al. 2016). Cellulose plays an increasingly significant role as a recyclable resource in many fields due to its nontoxicity, nonenvironmental pollution, and complete biodegradability (Xu et al. 2016, Bai et al. 2022). The share of carbon in the plant community is more than 50%, but its utilization rate is extremely low, resulting in a large waste of cellulose resources (Ghanbari et al. 2015). To improve its biological potential, several cellulosic biomasses have been extensively studied, mainly as raw materials for bioenergy production (Kralisch et al. 2010, Donato et al. 2019). The long chains of cellulose polymers are linked together to form a crystal structure of uniformly arranged microfibrils, making cellulose less susceptible to biodegradation (Ali et al. 2020).

The insect gut is colonized by a large diverse range of microorganisms, and symbiotic bacteria and fungi that detoxify the host (Lun et al. 2019, Nazni et al. 2019), provide nutrients (Luo et al. 2019), improve insect immunity (Lunsin et al. 2021), protect insects from pathogens and parasites (Lunsin 2021) and contribute to interspecies and intraspecific communication (Paniagua et al. 2018, Luo et al. 2019). Multiplant-feeding insects, such as termites, beetles, and grasshoppers, have efficient cellulose microbial transformation systems in their bodies (Kundu et al. 2019), and cellulase-producing intestinal bacteria digest lignocellulose into sugar, providing a major source of energy for insects (Hong et al. 2020). Both bacteria and fungi are capable of cellulase production, and bacterial cellulases usually have the advantages of wider use, stable reaction conditions, and short fermentation time for enzyme production (Zhao et al. 2003).

Grasshoppers are one of the most important agricultural phytophagous pests (Liu et al. 2007). Cellulose is a major component of the diet of grasshoppers, and the gut microbiota of grasshoppers plays an important role in the ability of insects to utilize cellulose-based food sources. Microorganisms contribute to the degradation of cellulose and may facilitate the digestion and absorption of other components (Ali et al. 2020). This potentially could be a tool for efficient cellulose catabolism. The presence of a large microbial resource in the gut of grasshoppers is a potential biomass energy transformer. Therefore, in this study, individual colonies capable of cellulase production were isolated from the intestines of *Atractomorpha sinensis*, *Trilophidia annulata*, *Sphingonotus mongolicus*, and *Calliptamus abbreviatus* using traditional microbial isolation techniques, and the strains were identified by combining morphological characteristics and phylogenetic analysis. The cellulose degradation rates of the isolates were further tested by DNS measurements. Our results may provide valuable microbial resources for improving the conversion efficiency of cellulosic biomass such as agricultural straw.

## Materials and Methods

### Insect Collection and Dissection

Four species of grasshoppers were collected from Baoding, Hebei Province, China, including *Atractomorpha sinensis*, *Trilophidia annulata*, *Sphingonotus mongolicus*, and *Calliptamus abbreviatus*. The grasshoppers were starved in an incubator at a temperature of 28 ± 0.5 °C and a relative humidity of 60 ± 5% for 3 days in the dark. Four grasshoppers of each species were randomly selected as one replicate, with a total of 3 replicates. The surfaces of the grasshoppers were disinfected with 75% alcohol and irradiated under UV light for 3–5 min and repeated 3 times to avoid external contamination (Morales-Jiménez et al. 2012). After dissection under aseptic conditions, the intestines were transferred to sterile centrifuge tubes and weighed, and added to the corresponding sterile centrifuge tubes containing 2 ml of sterile water for grinding at a mass to volume ratio of 1: 9.

### Isolation and Purification of Cellulose-Degrading Bacteria

Grasshopper intestinal flora was inoculated into LB (beef paste peptone) liquid culture medium at a proportion of 1%, and sterile water was added as a control. After inoculation, the culture was incubated in a thermostatic shaker at 37 °C and 150 r/min. When the bacterial liquid was turbid, the culture was diluted in a concentration gradient from 10^−1^ to 10^−6^ and 100 μl dilutions were applied to the CMC screening medium (carboxymethylcellulose sodium medium). Each medium was repeated 3 times. The cultures were incubated upside down at 37 °C for 24 h. The colonies grown were purified to single colonies and then subjected to subsequent experiments.

### Phenotypic Identification of Cellulose-Degrading Bacteria

Morphological characteristics such as color, shape, edge, projection, transparency, and luster of individual colonies were observed and described with reference to Shen and Chen (Shen and Chen, 2018).

### Molecular Identification of Cellulose-Degrading Bacteria

The purified bacterial strains were inoculated in 10 ml of LB liquid medium at 1%. After inoculation, the culture was incubated for 12 h at 37 °C with shaking at 200 rpm, and 1 ml of turbid bacterial suspension was used to extract the bacterial genome. For the extraction of bacterial genomic DNA, the E.Z.N.A. A soil DNA kit (Omega Bio-Tek, USA) was used to extract bacterial genomic DNA according to the manufacturer’s instructions. Total bacterial DNA extracted was used as a template using Taq DNA polymerase (Accurate, China), and 16S rDNA universal primers 27F (5’-AGAGTTTGATCCTGGCTCAG-3ʹ) and 1492R (5ʹ-GGTTACCTTGTTACGACTT-3ʹ) were used for PCR amplification. The total volume of PCRs was 25 μl, including 9.5 μl ddH_2_O, 12.5 μl 2 × Taq Mix, 1.0 μLg DNA (50 ng/μl), 1 μl primer 27F (10 μM) and 1 μl of primer 1492R (10 μM). The PCR reaction program was set to 94 s at 30 °C; 10 s at 98 °C, 30 s at 55 °C, and 30 cycles of 2 min at 72 °C. The PCR product quality was detected by electrophoresis on a 1% agarose gel to observe the size of the target bands. The purified PCR amplification products were sent to Bioengineering (Shanghai) Co. for sequencing.

After initial quality assessment, the sample sequences were compared with other 16S rDNA sequences in the NCBI database using BLAST (Basic Local Alignment Search Tool) (Korf et al. 2003). The best nucleic acid substitution model was selected in MEGA X using the Akira Pool Information Criterion (AIC) (Kumar et al. 2018), and phylogenetic analysis was performed using the maximum likelihood (ML) method with bootstrap = 1,000. Phylogenetic trees were visualized using FigTree v1.4.4 (Rambaut, 2010).

### Physiological and Biochemical Identification of Cellulose-Degrading Bacteria

Putative phenotypic bacterial identification of selected isolates was performed using standard microbiological methods, including Gram staining and biochemical tests for oxidase, V–P, and indole production (Zhang. 2021).

### Determination of Enzyme Activity

#### Glucose standard curve generation

The glucose standard curve was made as follows: 1.00 g of constant weight glucose was weighed and prepared as a 1.00 mg/ml glucose solution (glucose standard solution). Reagents were added to the test tubes as shown in Table 1. Dinitro salicylic acid reagent (2.50 ml) was added to each tube, which was shaken, boiledin a water bath for 5 min, and quickly cooled to room temperature with cold water. Then, the volume was adjusted to 10.00 ml with deionized water, and 3 parallel samples and one blank control were made for each tube. The glucose standard curve was plotted using the mean value of the OD value (540 nm) of the spectrophotometer as the vertical coordinate (Y) and the glucose content (mg) as the horizontal coordinate (X) (Association of Personal Historian. 2015).

**Table 1.** Glucose standard curve preparation

Tube number	*V* (glucose standard solution)/mL	*V* (distilled water)/mL	*m* (glucose)/mg	Tube number	*V (glucose standard solution)/mL2*	*V* *(distilled water)/mL3*	*m (glucose)/mg4*
1	0.00	2.00	0.00	6	1.00	1.00	1.00
2	0.20	1.80	0.20	7	1.20	0.80	1.20
3	0.40	1.60	0.40	8	1.40	0.60	1.40
4	0.60	1.40	0.60	9	1.60	0.40	1.60
5	0.80	1.20	0.80	10	1.80	0.20	1.80

#### Determination of cellulase activity (CMCA)

The CMCA was determined by the DNS (dinitrosalicylic acid, 3, 5-dinitrosalicylic acid 0.63 g/L, potassium sodium tartrate 18.2 g/L, NaOH 2.1 g/L, phenol 0.5 g/L) colorimetric method. The crude enzyme solution was taken separately and put into test tubes, 1.5 ml of citric acid buffer (sodium citrate 1 g/L) dissolved in 1% CMC-Na (sodium carboxyl methyl cellulose) was added, and 2.5 ml of DNS was quickly added to the test tubes at 50°C for 30 min, boiled in a water bath for 5 min, and cooled to room temperature. Finally, 5.0 ml of deionized water was added to fix the volume to 10.0 ml. The OD value was determined at a wavelength of 540 nm. The OD value was measured (Zhang et al. 2021), and 3 parallel and one blank control were made for each strain to obtain the CMCA by a glucose standard curve.

The 3 parallel OD values of each strain were averaged, and the corresponding A values were calculated by substituting them into the glucose standard curve. Then, the enzyme activity was calculated according to the following equation.


$$\begin{array}{l} Cellulase\;activity\;(CMCA)\;(U/mL)\;=\;\;\;\frac{A*V_{1}*1,000}{M*T*V_{2}} \end{array}$$

A is the measured OD value calculated by substituting the standard curve equation for the glucose content (mg/ml), V_1_ is the total volume of reaction solution, 1,000 is the unit mg converted to μg, M is the molar mass of glucose (180 g/mol), T is the color development time (min), and V_2_ is the volume of crude enzyme liquid (ml). One unit (U) of enzyme activity is defined as the amount of enzyme that releases 1 µmol of reducing sugar equivalent to glucose per minute during the reaction.

### Determination of Filter Paper Glycosylase Enzyme Activity (FPA)

One milliliter of each prepared crude enzyme solution was placed into a test tube, and 1.5 ml of citric acid buffer was added. Then, the filter paper was cut into long strips of 1.0 cm × 6.0 cm, placed into the test tube, and preheated in a water bath at 50 °C for 2 min. The rest of the method was the same as that used for CMCA determination.

The 3 parallel OD values of each strain were averaged, and the corresponding A values were calculated by substituting them into the glucose standard curve. Then, the enzyme activity was calculated according to the following equation.


$$\begin{array}{l} Filter\;paper\;glycolytic\;enzyme\;activity\;(FPA)\;(U/mL)\;=\frac{A*V_{1}*1000}{M*T*V_{2}} \end{array}$$

A is the measured OD value calculated by substituting the standard curve equation for the glucose content (mg/ml), *V*_1_ is the total volume of reaction solution, 1,000 is the unit mg converted to μg, *M* is the molar mass of glucose (180 g/mol), *T* is the color development time (min), and *V*_2_ is the volume of crude enzyme liquid (ml). One unit (*U*) of enzyme activity is defined as the amount of enzyme that releases 1 µmol of reducing sugar equivalent to glucose per minute during the reaction.

## Results

### Isolation and Morphological Characterization of Cellulose-degrading Bacteria in the Gut of 4 Grasshopper Species

In this experiment, intestinal microorganisms of 4 grasshoppers were cultured and screened in 11 strains. Four strains were screened according to the species classification of *Trilophidia annulata* as follows: Y1, Y5, Y8, and Y9; 5 strains were isolated from *Atractomorpha sinensis*, named F5, F6, F7, F8, and F9; one strain was screened from *Sphingonotus mongolicus*, S1; and one strain was screened from *Calliptamus abbreviatus*, D1. The results of morphological identification of the strains are displayed in Table 2. The isolated bacteria exhibited different colony morphologies, colors, edges, and textures. Morphologically, Y5 was a pseudoradical strain, Y8 and F5 were irregular strains, and the rest were round strains. The color of the F6 strain is yellowish; the rest of the strains are creamy white; F6, F7, F8, F9, S1, and D1 have complete edges; Y1, Y8, and Y9 have erosion edges; Y5 has filamentous edge; and F5 has undulating edge. Texturally, Y1, Y5, Y8, and F5 are rough, and the rest of the strains are smooth.

**Table 2.** Morphological characteristics of each colony

Strain	Morphology	Color	Edge	Texture	Transparency
Y1	Round	White	Erosive	Rough	Opaque
Y5	Pseudoradical	White	Filiform	Rough	Opaque
Y8	Irregular	White	Erosive	Rough	Opaque
Y9	Round	White	Erosive	Smooth	Opaque
F5	Irregular	White	Wavy	Rough	Opaque
F6	Round	Yellowish	Complete	Smooth	Opaque
F7	Round	White	Complete	Smooth	Opaque
F8	Round	White	Complete	Smooth	Opaque
F9	Round	White	Complete	Smooth	Opaque
S1	Round	White	Complete	Smooth	Opaque
D1	Round	White	Complete	Smooth	Opaque

### Molecular Identification of Cellulose-Degrading Bacteria in the Gut of 4 Grasshoppers

The identity between the sequences of the 11 strains in the database and the known 16S rDNA sequences could reach 100% by BLAST. The strain splice sequence lengths and the genus-level classification of the highest homology sequences of each strain are shown in Table 3. BLAST results showed that 4 strains, Y1, Y5, Y8, and Y9, isolated from the gut of *Trilophidia annulata* had the highest identity with the genus *Bacillus*, and 5 strains isolated from the gut of *Atractomorpha sinensis*, of which F5 had the highest identity with the genus *Bacillus* and F6, F7, F8, and F9 had the highest identity with the genus *Aeromonas F5*. One strain each isolated from the gut of *Sphingonotus mongolicus* and *Calliptamus abbreviates* showed the highest identity with *Klebsiella*. The DNA sequences of strains D1, S1, F5, F6, F7, F8, F9, Y1, Y5, Y8, and Y9 were uploaded to GenBank under the numbers OQ747965, OQ747966, OQ747967, OQ747968, OQ747969, OQ747970, OQ747971, OQ747972, OQ747973, OQ747974, and OQ747975, respectively.

**Table 3.** Splicing sequence information of each strain

Strain	Sequence lengths (bp)	Genus-level classification of homologous sequences	DNA sequences
Y1	1,425	*Bacillus*	OQ747972
Y5	1,424	*Bacillus*	OQ747973
Y8	1,424	*Bacillus*	OQ747974
Y9	1,425	*Bacillus*	OQ747975
F5	1,297	*Bacillus*	OQ747967
F6	1,358	*Atractomorpha*	OQ747968
F7	1,418	*Atractomorpha*	OQ747969
F8	1,416	*Atractomorpha*	OQ747970
F9	1,417	*Atractomorpha*	OQ747971
S1	1,413	*Klebsiella*	OQ747966
D1	1,415	*Klebsiella*	OQ747965

**Fig. 1.**
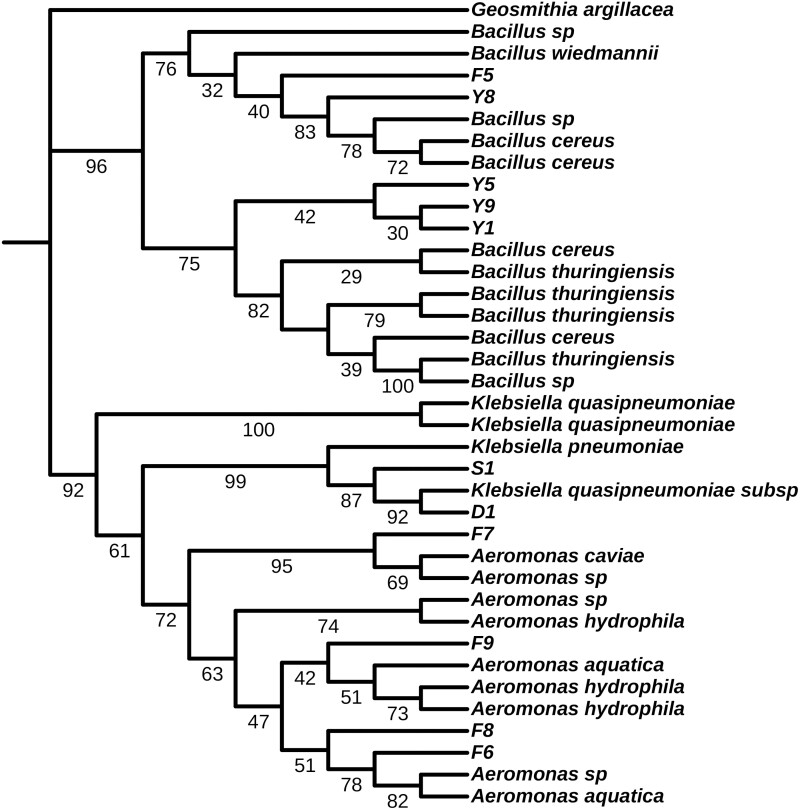
Phylogenetic tree. F5, F8, Y5, Y9, Y1 are the same genus, belonging to *Bacillus*. D1 and S1 aer the same genus *Klebsiella*, F7, F9, F8, F6 are the same genus,belonging to Aeromonas.

The topology of the ML phylogeny based on the 16S rDNA gene sequence of cellulose-degrading bacteria is shown in Fig. 1. All strains clustered in 3 main branches, including the *Bacillus*, *Aeromonas*, and *Klebsiella*. Specifically, the phylogenetic tree showed that F7 clustered with *Aeromonas caviae*, F6 and F8 clustered with *Aeromonas aquatica*, F9 clustered with *Aeromonas hydrophila*, F8, F9, F6, and F7 belonged to *Aeromonas*, and S1 and D1 clustered with *Klebsiella quasipneumoniae* and belonged to *Klebsiella*. Among them, Y1, Y5, and Y9 clustered with *Bacillus thuringiensis*, F5 and F8 clustered with *Bacillus cereus*, and Y1, Y5, Y8, Y9, and F5 belonged to *Bacillus*.

### Physiological and Biochemical Identification of Cellulose-Degrading Bacteria in the Intestines of 4 Grasshoppers

Based on the above molecular identification results, we identified the physiological and biochemical characteristics of the strains according to their genera, and the results are shown in Tables 4–6. Gram staining results showed that Y1, Y5, Y8, Y9, and F5 were positive; D1, S1, F6, F7, F8, and F9 were negative. The biochemical indices showed that Y1, Y5, Y8, Y9, and F5 were all consistent with the phylogenetic analysis and morphological identification of *Bacillus*; F6, F7, F8, and F9 were consistent with the phylogenetic analysis and morphological identification of *Aeromonas*; and D1 and S1 were consistent with the phylogenetic analysis and morphological identification of *Klebsiella*.

**Table 4.** Physiological and biochemical reaction results of *Bacillus*

Strain reaction	Y1	Y5	Y8	Y9	F5
Exposure to enzymes	+	+	+	+	+
Kinetic test	−	−	−	−	−
Nitrate reduction	+	+	+	+	+
Glucose	+	+	+	+	+
V.P	+	+	+	+	+
Mannitol acid production	−	−	−	−	−
Gelatin decomposition	+	+	+	+	+
Citrate	−	−	−	−	−
Hydrogen sulfide test	−	−	−	−	−
Indole test	−	−	−	−	−
Methyl red test	+	+	+	+	+

**Table 5.** Physiological and biochemical reaction results of *Aeromonas*

Strain reactive	F6	F7	F8	F9
Glucose	+	+	+	+
Contact enzyme	+	+	+	+
Sucrose	+	+	+	+
Heptaoside	+	+	+	+
arabinose	+	+	+	+
Urea	−	−	−	−
Salicin	+	+	+	+
Inositol	−	−	−	−
Gelatin	+	+	+	+
Nitrate reduction	+	+	+	+

**Table 6.** Physiological and biochemical reaction results of *Klebsiella*

Strain reactive	S1	D1
Glucose	+	+
Maltose	+	+
Kinetic test	−	−
Methyl Red	+	+
V.P	+	+
Mannitol	+	+
Sucrose	+	+
Lactose	+	+

### Analysis of the Degradation Ability of Cellulose-Degrading Bacteria in the Intestine of Grasshoppers

**Fig. 2.**
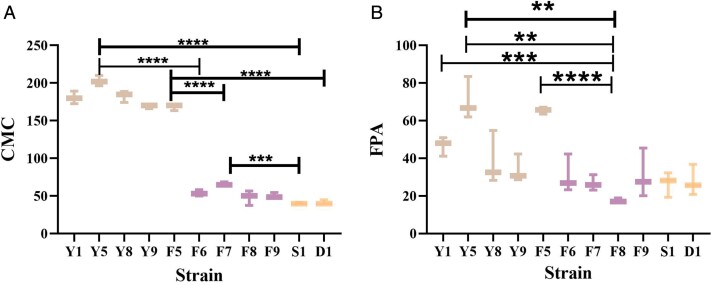
Determination of enzyme activity. Y1, Y5, Y8, Y9, F5 belong to *Bacillus*, F5, F6, F7, F8, F9 belong to Aeromonas.S1 and D1 belong to *Klebsiella* The horizontal coordinate is the strain name and the vertical coordinate is the enzyme activity, the left graph is the CMC enzyme activity, and the right graph is the FPA enzyme activity. ** represents a significant difference, *** as well as **** represents a highly significant difference. Due to the large number of strains, only highly significant differences are shown in this figure.

Eleven strains of cellulose-degrading bacteria were made into crude enzyme solutions, and then the cellulase activity was tested. The standard curves for calculating the degradation rates are shown in Supplementary Material 1. The results showed that the CMCA of the 11 strains was consistent with the results of FPA (filter paper glycosylase enzyme activity, hydrolyzes filter paper to produce reducing sugars), but the CMCA was more representative in Fig. 2. Overall, the CMCA indicated that the enzyme activity of *Bacillus* was significantly higher than that of *Aeromonas* and *Klebsiella*. The cellulase activity of *Bacillus* was higher than that of *Klebsiella*. Specifically, the lowest enzyme activity in the *Bacillus strain* (F5) was significantly higher than that in the *Aeromonas* (F7) and *Klebsiella* strains. The enzyme activity of F7 in *Aeromonas* was significantly higher than that of *Klebsiella* S1, while the other strains did not differ significantly. Similar results were found for the filter paper glycosidase activity, which was significantly higher in *Bacillus* than in *Aeromonas* and *Klebsiella*. In contrast to the CMCA, FPA showed that all strains of *Bacillus* were not significantly different from the 2 strains of *Klebsiella*.

## Discussion

Insects rely on intestinal microorganisms to digest cellulose for its nutritional value, in particular, terminating, grasshoppers, and aspen, feed on cellulose-rich plants, and their ability to degrade cellulose is approximately 20–100%, which is 30–40% higher than that of large herbivores (Zhang et al. 2021). These insects contain diverse cellulose-degrading bacteria and are a resource pool of cellulose-degrading bacteria, including *Enterobacteriaceae*, *Bacteroides*, *Staphylococcus*, *Streptococcus*, and *Bacillus*. Plants are rich in cellulose, a polymeric fraction that is widely available, and has high potential for industrial applications such as biogas and ethanol production; however, cellulose degradation efficiency issues greatly limit the utilization of this biomass energy source. We found that insect gut microorganisms are capable of producing cellulose-degrading enzymes, the production, and secretion of which is an important feature of certain bacterial strains and can be a tool for efficient cellulose breakdown. In this study, we first isolated and characterized 4 species of cellulose-degrading bacteria from the gut of grasshoppers. These bacteria were shown to degrade cellulose when cultured on CMC media alone. After measuring the cellulose degradation rate of each bacterial strain by DNS, it was found that all strains could effectively degrade cellulose on the medium, and the degradation efficiency of *Bacillus* was relatively significant. Taken together, these results demonstrate that the gut bacteria of grasshoppers can help grasshoppers digest and absorb cellulose, providing a valuable microbial resource for addressing cellulose biotransformation.

Traditional microbial isolation techniques and ideal growth conditions play a key role in the identification of efficient bacterial isolates. Physiological and biochemical characterization as well as 16S rDNA sequences are traditional methods for strain identification (Fan et al. 2012, Rong et al. 2006, Yang et al. 2014). In previous studies, *Bacillus cereus* was also isolated from the locust gut by traditional isolation techniques (Xu et al. 2016). Domestic studies on grasshopper gut microorganisms have been less reported, mainly focusing on some mammals, in which cellulose-degrading bacteria with high enzymatic activity were isolated from the feces of Bactrain camels (Jing et al. 2019) and yaks (Mao et al. 2019). However, most of these studies involved *Bacillus* and *Enterobacter* as well as *Klebsiella*. This is consistent with the results of our study. *Klebsiella*, *Aeromonas*, and *Bacillus* isolated in this study were able to survive on cellulose (CMC) as the only carbon source, suggesting that cellulose can be normally degraded and utilized by these bacteria in vitro. Other bacteria such as *Pseudomonas* and *Serratia* have been isolated from other phytophagous insects that have cellulolytic activity. *Klebsiella* and *Bacillus* in the termite gut have strong cellulose degradation capacity (Xie et al. 2019), and this study found a similar phenomenon. In grasshoppers, 2 species of *Bacillus* were screened, and it was confirmed that *Bacillus* had a strong cellulose digestion ability (Bai et al. 2022), which is consistent with our findings. The physiological role of these bacteria in the gut of grasshoppers may be similar to that of intestinal commensal bacteria in other phytophagous insects. In this study, *Bacillus* was isolated from the intestine of grasshoppers, and *Bacillus* also were isolated from the intestine of *Schistocerca gregaria*, *Bacillus* was shown to have the function of promoting the secretion of digestive enzymes in the intestine, which can improve the digestion and absorption ability of the body and enhance immunity (Ren et al. 2014, Fooladi et al. 2013).

Cellulolytic bacteria from termites gave *Klebsiella Michigan* an optimal cellulase activity of 35.6 U/ml at pH 7 (Olowomofe et al. 2019). The CMCA of the bacteria isolated from *Odontotermes formosanus Shiraki* reached the maximum (20.8 U/ml). The cellulolytic activity of *Pseudomonas* and *Bacillus* isolated from termites of Bhitarakanika, Odisha reached 66.9 U/ml, 156 U/ml, respectively. (Thatoi et al. 2012). The bacteria isolated and screened from the intestines of 4 species of grasshoppers in this study reached a maximum CMCA of 202.68 U/ml and a maximum FPA of 70.66 U/ml. In summary, the bacterial enzyme activity isolated from the grasshopper gut was higher than the cellulase activity of other insect gut bacteria.

Through comparative analysis of 4 species of grasshoppers, we found that different grasshoppers had different symbiotic bacterial populations in their intestines and extraordinarily different digestive capacities. In this study, *Sphingonotus mongolicus* and *Calliptamus abbreviations* are distributed in northeastern and northern China, and their similar geographic environments lead to similar feeding habits. The bacteria screened out by both *Sphingonotus mongolicus* and *Calliptamus abbreviations* were identified as *Klebsiella*, which may be related to their feeding habits. Eight strains of bacteria were isolated and purified from *Locusta migratoria* intestinal bacteria cultured under laboratory conditions by Zhao (Zhao et al. 2008), and the host bacteria were dominated by *Klebsiella* and *Enterobacter*. In contrast, the intestinal bacteria of *Schistosoma japonicum*, which feeds on plant leaves and stems, were dominated by *Enterobacter* and *Streptococcus*. The results of the intestinal bacterial analysis also showed that grasshoppers are adapted to different types of feed by regulating the cellulose-degrading bacterial species in the intestine (Su et al. 2014).

In conclusion, similar to other herbivorous insects, the gut microbiota of grasshoppers is clearly important for plant degradation. These bacteria can degrade cellulose to varying degrees to produce secondary metabolites, and the high degradation capacity of *Bacillus* in particular highlights the important role of gut bacteria in cellulose degradation. It is important to note that our current work is insufficient, and in-depth studies should focus on testing the physiological role in vivo as well as environmental tests outside the strain and elucidating the metabolic pathways of these bacteria in cellulose degradation. Our study isolated, characterized and identified 11 bacterial strains, 3 cellulose-degrading genera, and showed that *Bacillus* had the highest cellulose degradation properties. Through further evaluation, these identified genera can be developed into effective engineered bacteria that can provide a theoretical and practical basis for solving cellulosic biomass conversion problems.

## Supplementary Material

iead101_suppl_Supplementary_Materials
