# Supplementary material for: Isolation and characterization of intestinal bacteria associated with cellulose degradation in grasshoppers (Orthoptera)
Source: J Insect Sci. 2023 Nov 24;23(6):7. doi: 10.1093/jisesa/iead101 (PMC10676120; doi:10.1093/jisesa/iead101)
Supplement: iead101_suppl_Supplementary_Materials [file iead101_suppl_supplementary_materials.docx]

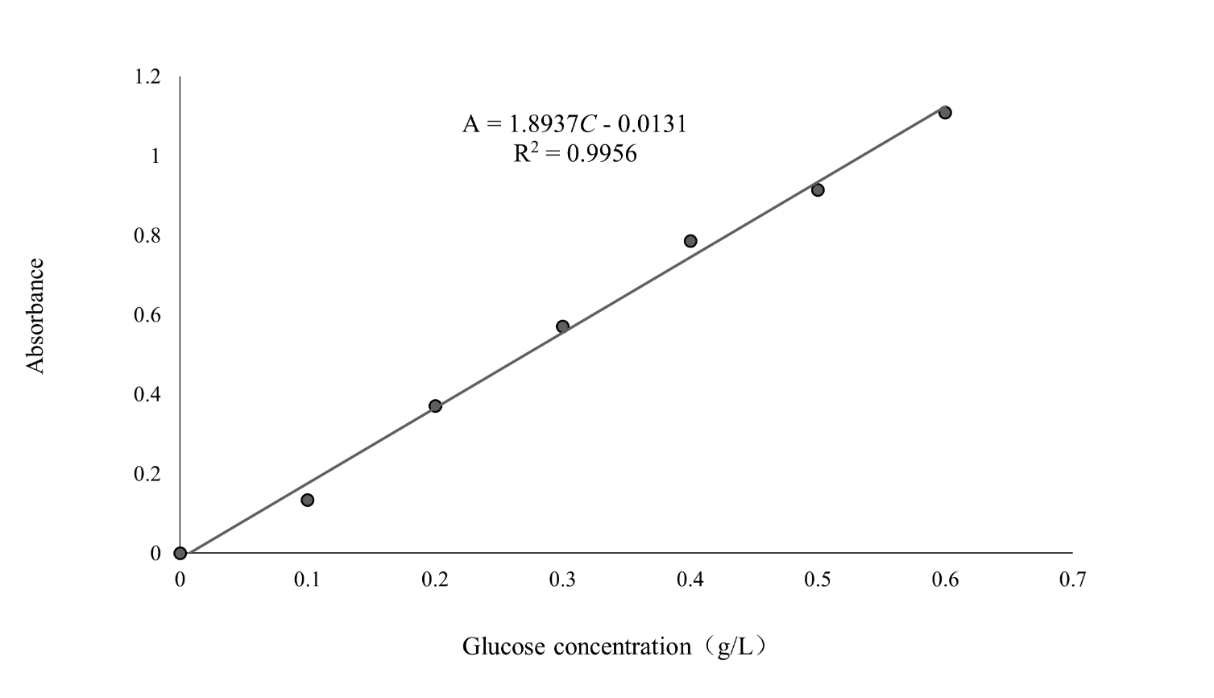


**Supplemental materials 1**: Glucose standard curve

Figure 1 shows the standard glucose curve with the equation A=1.8937C-0.0131and R^2^=0.9978, which can be used to estimate the enzyme activity. A is the vertical coordinate in the graph, C is the horizontal coordinate in the graph. A is the measured OD value calculated by substituting the standard curve equation for the glucose concentration (g/mL).
